# RIPK1 polymorphisms and expression levels: impact on genetic susceptibility and clinical outcome of epithelial ovarian cancer

**DOI:** 10.1186/s12935-023-03139-7

**Published:** 2023-11-23

**Authors:** Xuedong Wang, Kui Deng, Jing Tao, Juan Zou, Yiting Du, Li Dai

**Affiliations:** 1 Key Laboratory of Birth Defects and Related Diseases of Women and Children (Sichuan University), Ministry of Education, West China Second University Hospital, Chengdu, Sichuan China; 2 National Center for Birth Defect Monitoring, West China Second University Hospital, Sichuan University, Chengdu, Sichuan China; 3 Department of Pathology, West China Second University Hospital, Sichuan University, Chengdu, Sichuan China; 4 Medical Big Data Center, Sichuan University, Chengdu, Sichuan China

**Keywords:** Epithelial ovarian cancer (EOC), *RIPK1*, Polymorphisms, Expression, Susceptibility, Prognosis

## Abstract

**Background:**

The aim of this study was to explore the associations of *RIPK1* polymorphisms, plasma levels and mRNA expression with susceptibility to epithelial ovarian cancer (EOC) and clinical outcome.

**Methods:**

Three hundred and nineteen EOC patients included in a 60-month follow-up program and 376 controls were enrolled. Two tag SNPs (rs6907943 and rs9392453) of *RIPK1* were genotyped using polymerase chain reaction (PCR)-restriction fragment length polymorphism (RFLP) method. Plasma levels of RIPK1 and *RIPK1* mRNA expression in white blood cells were determined by ELISA and qPCR, respectively.

**Results:**

For rs9392453, significantly increased EOC risk was found to be associated with C allele (*P* = 0.002, OR = 1.49, 95%CI  1.15–1.92), and with CT/CC genotypes in the dominant genetic model (*P* = 0.006, OR = 1.54, 95%CI  1.12–2.08). CC haplotype (rs6907943-rs9392453) was associated with increased EOC susceptibility. CC genotype of rs6907943 and CT/CC genotypes of rs9392453 were associated with early onset (age ≤ 50 years) of EOC (OR = 2.5, 95%CI  1.03–5.88, and OR = 1.64, 95%CI  1.04–2.63, respectively). AC genotype of rs6907943 was associated with better overall survival of EOC patients in the over-dominant genetic model (*P* = 0.035, HR = 0.41, 95%CI  0.18–0.94). Multivariate survival analysis identified the AC genotype of rs6907943 as an independent protective factor for survival of early onset patients (*P* = 0.044, HR = 0.12, 95%CI  0.02–0.95). Compared to controls, significantly increased plasma levels of RIPK1 and reduced *RIPK1* mRNA expression were observed in patients.

**Conclusions:**

Our results suggest that tag SNPs of *RIPK1*, increased plasma levels of RIPK1 protein and reduced *RIPK1* mRNA expression in white blood cells, may influence the susceptibility to EOC. SNP rs6907943 may be a useful marker to distinguish EOC patients with high risk of death.

**Supplementary Information:**

The online version contains supplementary material available at 10.1186/s12935-023-03139-7.

## Background

Ovarian cancer is the eighth most common cancer in females and is the leading cause of death among the malignancies of female reproductive system, with an estimated 313,959 new cases and 207,252 deaths worldwide in 2008 [1]. Epithelial ovarian cancer (EOC) is the most common type of ovarian cancer. These characteristics for the course of most ovarian cancers, late-stage disease at diagnosis and acquired resistance to chemotherapy, limit the possibility of effective cure. The 5-year overall survival rate remains 42–26% for advanced stages (FIGO stages III and IV) [2]. Yet, pathogenesis of EOC is still not fully understood, known risk factors include family history of ovarian or breast cancer, never pregnant, history of hormone replacement therapy [3]. EOC is a multifactorial disease and genetic susceptibility has been identified in numerous studies. Females that inherit a deleterious mutation in *BRCA1* or *BRCA2* gene have a high life-time risk for EOC and to some extent, increased risks for fallopian tube and peritoneal cancer [4]. The potential roles of genetic polymorphisms as predictive or prognostic biomarkers in ovarian cancer have been widely studied [4–6].

The receptor (TNFRSF)-interacting serine-threonine kinase 1 (RIPK1) was first identified as a death domain-containing kinase which is recruited to the intracellular death domains of FAS and TNF receptor 1 (TNFR1) upon ligand stimulation [7]. As crucial cellular processes, inflammation and cell death control organismal homeostasis and viability. Emerging evidence indicates that RIPK1 is a key upstream regulator which controls inflammatory signaling and the activating of multiple cell death pathways, including apoptosis and necroptosis [8]. In response to stimuli such as TNF and ligands of the Toll-like receptor (TLR) family, RIPK1 regulates inflammatory signaling in both kinase-dependent and -independent manners [9, 10]. RIPK1 has also been implicated in the regulation of apoptosis as well as necroptosis [11–13]. The ubiquitination state of RIPK1 determines whether it function as a pro-survival scaffold molecule or a kinase that promote cell death. RIPK1 decorated with ubiquitin chains linked through K63 (lysine 63) of ubiquitin promotes the activation of mitogen-activated protein kinases (MAPKs) and NF-κB, resulting the expression of pro-survival genes [14]. While K63 ubiquitination of RIPK1 and TNF-induced NF-κB activation are reduced, RIPK1 switches its function to that of promoting cell death [11, 14]. The RIPK family, which share a homologous N-terminal kinase domain but have different recruitment domains, collaborates with death receptor proteins to regulate cell death. RIPK3 kinase functions with RIPK1 at the crossroads of apoptosis, necroptosis, and cell survival [14–16]. Caspase-8-mediated cleavage of RIPK1 and RIPK3 will trigger the caspase cascade and induce apoptosis, while the inhibition of caspase-8 or knockout of Fas-associated death domain (FADD) will abrogate the apoptosis signal to favor necroptosis [17–19].

Polymorphisms of *RIPK1* gene have been reported to be associated with multiple myeloma, diffuse large B cell lymphoma, colorectal cancer, and childhood leukemia [20–23]. However, little is known about the effects of *RIPK1* gene on the susceptibility and prognostic outcome of EOC. Accordingly, the present study analyzed the influence of *RIPK1* tag SNPs, plasma levels of RIPK1 and *RIPK1* mRNA expression on the susceptibility to EOC and prognosis of patients with EOC.

## Methods

### Study subjects

A hospital-based case-control study was conducted including 319 unrelated EOC patients (mean ± SD, 50.21 ± 10.34 years) derived from the West China Second University Hospital of Sichuan University. The diagnosis of EOC was confirmed in all cases by histological examination of tissue from resected specimens. Clinical data were abstracted from patients’ medical records. The patients were followed up by telephone calls every 6 months for 3 years and every 1 year thereafter from the date of diagnosis to cancer-specific death or the date of last follow-up whichever came first. Those patients who had previous cancer, previous radiotherapy or chemotherapy, and metastasized cancer from other or unknown origins were excluded. The control group consisted of 376 healthy subjects (mean ± SD, 50.88 ± 10.88 years) from a routine health survey in the same hospital. Control subjects were genetically unrelated individuals and those with any personal or family history of cancer or other serious disease were intentionally excluded. The level of plasma RIPK1 and expression of RIPK1 mRNA were analyzed in 66 patients (mean ± SD, 51.17 ± 8.92 years) and 75 controls (mean ± SD, 50.83 ± 10.81 years). All subjects were Han females living in Sichuan province of southwest China. This study was approved by the ethics committee of West China Second University Hospital. All subjects gave written informed consent to participate. Patients’ characteristics are summarized in Table 1.

**Table 1:** Characteristics of studied subjects

Characteristics	EOC	Controls
*N*	319	376
Age, years (Mean ± SD)	50.21 ± 10.34	50.88 ± 10.88
Histology, no. (%)		
Serous-papillary	205 (64.3)	–
Endometrioid	18 (5.6)	–
Mucinous	14 (4.4)	–
Clear cell	27 (8.5)	–
Mixed and other	55 (17.2)	–
FIGO stage, no. (%)		
I–II	97 (31.2)	–
III–IV	222 (68.8)	–
Tumor grade, no. (%) ^a^		
G1	24 (8.3)	–
G2	38 (13.1)	–
G3	227 (78.5)	–

^a^30 patients with missing tumor grade value removed

### SNP selection, DNA isolation and genotyping

By using the algorithm-Tagger-pairwise Tagging from HapMap, tag SNPs of *RIPK1* gene (rs6907943 located in intron 2 and rs9392453 located in intron 5, respectively) were picked out for population CHB. Genomic DNA of each individual was extracted from 200 µl EDTA-anticoagulated peripheral blood samples by a DNA isolation kit from Bioteke (Peking, China) and the procedure was performed according to the manufacturer’s instructions. Genotyping was performed using the PCR-RFLP method. Primers were established with the PIRA PCR designer (http://cedar.genetics.soton.ac.uk/public_html/primer2.html) [24]. The primer sequences, corresponding endonucleases and reaction conditions are shown in Table 2. For each SNP, three subjects with different genotype were performed the DNA sequencing analysis to confirm the results genotyped by PCR-RFLP. About 10% of the samples were randomly selected to perform the repeated assays and the results were 100% concordant.

**Table 2:** Primer sequences and reaction conditions for genotyping two tag SNPs of *RIPK1* gene

SNP	Primer sequence	Ta (℃)	Endonuclease	Product size (bp)
rs6907943	F: GTGTTTGTTTGCAGCTCGTTAGCCT	62	*MnlI*	A: 120
	R: CAGGTGTTGGAGTTCAGCCTGG			C: 33 + 87
rs9392453	F: ACAAGCCCCACTTCAGGTTTGGT	62	*MspI*	T: 209
	R: CTGAACACACAGAAGCACTGGAATCC			C: 27 + 182

### Plasma RIPK1 and RIPK1 mRNA determination

For quantitative determination of RIPK1, peripheral blood from 66 patients and 75 controls was collected into vacutainer tubes containing EDTA-anticoagulant. Samples were centrifuged at 3000×g for 10 min and plasma was collected and stored at −80 ℃ until use. Plasma levels of RIPK1 were measured using commercially available ELISA kits (Ucsn Life, Houston City, TX) according to the manufacturer’s instructions. Developed color reaction was measured as OD450 units on a multimode microplate reader (TECAN Infinite M200, Switzerland). The plasma concentration of RIPK1 was determined using standard curve constructed with the kit’s standards over the range of 156 − 10,000 pg/ml. The minimum detectable dose of RIPK1 was typically less than 57 pg/ml.

Total RNA was extracted and purified from blood samples using TRIzol^®^ Reagent (Life Technologies, USA) according to the manufacturer’s protocol. Reverse transcription-PCR (RT-PCR) were performed by one-step RT-PCR kit (BIONEER, South Korea) according to the manufacturer instructions. Quantitative real-time PCR (qPCR) was carried out using SYBR green PCR Master Mix (Roche, Switzerland) and samples were amplified in a thermocycler as follows: 95 ℃ for 10 min, 49 cycles of 95 ℃ for 15s and 60 ℃ for 1 min. After PCR, melting curve analysis started with denaturation at 95 ℃ for 15s, followed by a temperature increase from 60 ℃ to 95 ℃ at 0.5 ℃/step with a 5s stop between each step, during which fluorescence was acquired from SYBR channel. The sequence of primer pairs for *RIPK1* and beta-actin (*ACTB*) gene are as follows: F: 5′-CTGGGCTTCACACAGTCTCA-3′, R: 5′-GTCGATCCTGGAACACTGGT-3′ and F: 5′-TGACGTGGACATCCGCAAAG-3′, R: 5′-CTGGAAGGTGGACAGCGAGG-3′, respectively. Data were normalized for *ACTB* expression using comparative threshold cycle method. Triplicate Ct values were averaged and the relative expression levels were determined as 2^–ΔΔCt^.

### Statistical analysis

Data were analyzed using SPSS for Windows software package version 13.0 (SPSS Inc., Chicago, IL, USA). Genotype frequencies of these two tag SNPs were obtained by directed counting. Hardy-Weinberg equilibrium was evaluated by chi-square test. Odds ratio (OR) and respective 95% confidence intervals (CI) were reported to evaluate the effects of any difference. Probability values of 0.05 or less were regarded as statistically significant in patients with EOC compared to controls. Genotypic association test in a case-control pattern assuming codominant, dominant, recessive, or over-dominant genetic models was performed using SNPstats [25]. The linkage disequilibrium (LD) and haplotypes between these two SNPs were analyzed by the SHEsis software platform [26]. The plasma RIPK1 levels and *RIPK1* mRNA expression between patients and controls were compared using the independent samples *t* test. Their expression levels among patients with different SNP genotypes were compared using the Kruskal-Wallis test.

Kaplan-Meier plots and the log-rank test were used to evaluate the association between *RIPK1* tag SNP and outcome from the date of primary diagnosis until death. Multivariate survival analysis was carried out by Cox regression analysis adjusted by the effect of age, FIGO stage, histological type, and tumor grade as well-established or widely discussed prognostic factors. Hazard ratio (HR) and 95%CI were calculated from the Cox regression model. The influence of *RIPK1* genotypes on the overall survival of different patients stratified by age, FIGO stage, histological type, and tumor grade was also analyzed.

## Results

These two tag SNPs of *RIPK1* gene were successfully genotyped in 319 patients with EOC and 376 control subjects. Three genotypes of each SNP were identified and the genotypes were confirmed by the DNA sequencing analysis. All observed genotype frequencies in both patients and controls were in agreement with that expected under the Hardy-Weinberg equilibrium. Genotype and allele frequencies of *RIPK1* tag SNPs in patients and controls are shown in Table 3. The results of genotype are shown in Additional file 1. Table S1. Significant difference in genotype and allele distribution of rs9392453 were observed between EOC patients and controls (*P* = 0.006, OR = 1.54, 95%CI  1.12–2.08 for genotype distribution in the recessive genetic model; and *P* = 0.002, OR = 1.49, 95%CI =1.15–1.92 for allele distribution, respectively). No significant difference was observed for the distribution of allele or genotype of rs6907943 between patients and controls.

**Table 3:** Distribution of the *RIPK1* tag SNPs among cases and controls and their associations with EOC risk

Model	Genotype	rs6907943				Genotype	rs9392453			
		EOC n (%)	Controls n (%)	OR (95% CI)	*P *value		EOC n (%)	Controls n (%)	OR (95% CI)	*P *value
Codominant	AA	168 (52.7%)	186 (49.5%)	1	0.7	TT	179 (56.1%)	249 (66.2%)	1	**0.01**
	AC	123 (38.6%)	155 (41.2%)	0.88 (0.64-1.20)		CT	117 (36.7%)	113 (30.1%)	1.45 (1.04-2)	
	CC	28 (8.8%)	35 (9.3%)	0.88 (0.52-1.52)		CC	23 (7.2%)	14 (3.7%)	**2.27 (1.15-4.55)**	
Dominant	AA	168 (52.7%)	186 (49.5%)	1	0.4	TT	179 (56.1%)	249 (66.2%)	1	**0.006**
	AC/CC	151 (47.3%)	190 (50.5%)	0.88 (0.65-1.19)		CT/CC	140 (43.9%)	127 (33.8%)	**1.54 (1.12-2.08)**	
Recessive	AA/AC	291 (91.2%)	341 (90.7%)	1	0.8	TT/CT	296 (92.8%)	362 (96.3%)	1	**0.042**
	CC	28 (9.1%)	35 (9.3%)	0.93 (0.56-1.59)		CC	23 (7.2%)	14 (3.7%)	**2.00 (1.02-4)**	
Overdominant	AA/CC	196 (61.4%)	221 (58.8%)	1	0.48	TT/CC	202 (63.3%)	263 (70%)	1	0.07
	AC	123 (38.6%)	155 (41.2%)	0.89 (0.66-1.22)		CT	117 (36.7%)	113 (30%)	1.35 (0.98-1.86)	
	Allele					Allele				
	A	459 (71.5%)	527 (70.1%)	1	0.45	T	475 (74.5%)	611 (81.2%)	1	**0.002**
	C	179 (28.1%)	225 (29.9%)	0.91 (0.72-1.15)		C	163 (25.5%)	141 (18.8%)	**1.49 (1.15-1.92)**	

Bold value Indicates a significant difference at the 5% level

No LD was observed between these two SNPs in our data (D’ = 0.33, *r*^2^ = 0.08). We further analyzed four haplotype combinations and found significant difference in the distribution of the haplotype frequencies between patients and controls (Table 4). Haplotype CC (rs6907943-rs9392453) was significantly associated with increased EOC risk.

**Table 4:** Haplotype frequencies of the *RIPK1* gene in patients and controls

Haplotypes	Frequency		OR (95% CI)	*P* Value
rs6907943-rs9392453	EOC	Controls		
AC	56.68 (0.089)	86.52 (0.115)	0.75 (0.53–1.07)	0.11
AT	402.32 (0.631)	440.48 (0.586)	1.21 (0.97–1.5)	0.09
CC	106.32 (0.167)	54.48 (0.072)	**2.56 (1.81–3.62)**	**4.56E-8**
CT	72.68 (0.114)	170.52 (0.227)	**0.44 (0.33–0.59)**	**3.52E-8**

Bold value Indicates a significant difference at the 5% level

Genotype distributions of *RIPK1* tag SNPs were analyzed in different groups stratified by EOC patients’ characteristics including age, FIGO stage, histological type and tumor grade (Table 5). For both rs6907943 and rs9392453, no significant difference between EOC patients stratified by FIGO stage, histological type or tumor grade was observed. CC homozygote of rs6907943 and allele C carriers (CT/CC genotypes) of rs9392453 were associated with a significantly increased EOC risk for young females (age ≤ 50 years) compared to old females (age > 50 years) (OR = 2.5, 95%CI = 1.03–5.88 for rs6907943, and OR = 1.64, 95%CI  1.04–2.63, respectively). The present results suggested that these two tag SNPs of *RIPK1* gene were associated with early onset of EOC.

**Table 5:** Association between the *RIPK1* tag SNPs and EOC patient’s characteristics

Characteristics	rs6907943						rs9392453					
	Genotype			OR (95% CI)^**a**^			Genotype			OR (95% CI)^**a**^		
	AA	AC	CC	Dominant (AA *vs.* AC/CC)	Recessive (AA/AC *vs.* CC)	Overdominant (AA/CC *vs.* AC)	TT	CT	CC	Dominant (TT *vs.* CT/CC)	Recessive (CT/TT *vs.* CC)	Overdominant (CT *vs.* CC/TT)
Age												
≤50	82 (49.1%)	65 (38.9%)	20 (12%)	1.35 (0.85-2.13)	**2.5 (1.03-5.88)**	1.02 (0.64-1.64)	85 (50.9%)	67 (40.1%)	15 (9%)	**1.64 (1.04-2.63)**	1.89 (0.76-4.76)	1.43 (0.88-2.27)
>50	86 (56.6%)	58 (38.2%)	8 (5.3%)				94 (61.8%)	50 (32.9%)	8 (5.3%)			
FIGO stage												
I-II	52 (53.6%)	36 (37.1%)	9 (9.3%)	0.64 (0.35-1.15)	0.88 (0.31-2.46)	0.65 (0.35-1.2)	57 (58.8%)	32 (33%)	8 (8.2%)	0.70 (0.39-1.26)	1.37 (0.47-4.00)	0.62 (0.33-1.15)
III-IV	116 (52.2%)	87 (39.2%)	19 (8.6%)				122 (55%)	85 (38.3%)	15 (6.8%)			
Histological type												
Serous	117 (56%)	74 (35.4%)	18 (8.6%)	1.55 (0.84-2.85)	0.90 (0.31-2.63)	1.63 (0.88-3.04)	117 (56%)	77 (36.8%)	15 (7.2%)	0.98 (0.53-1.82)	1.21 (0.39-3.79)	0.92 (0.49-1.75)
Non-serous	51 (46.4%)	49 (44.5%)	10 (9.1%)				62 (56.4%)	40 (36.4%)	8 (7.3%)			
Tumor grade b												
G1-G2	32 (51.6%)	24 (38.7%)	6 (9.7%)	0.97 (0.5-1.89)	1.01 (0.32-3.17)	0.97 (0.79-1.92)	36 (58.1%)	23 (37.1%)	3 (4.8%)	1.06 (0.54-2.09)	2.72 (0.64-11.55)	0.81 (0.41-1.62)
G3	124 (54.6%)	84 (37%)	19 (8.4%)				128 (56.4%)	81 (35.7%)	18 (7.9%)			

Bold value Indicates a significant difference at the 5% level

^a^Adjusted for age, FIGO stage, histological type, and tumor grade

^b^30 patients with missing tumor grade value removed

**Fig. 1:**
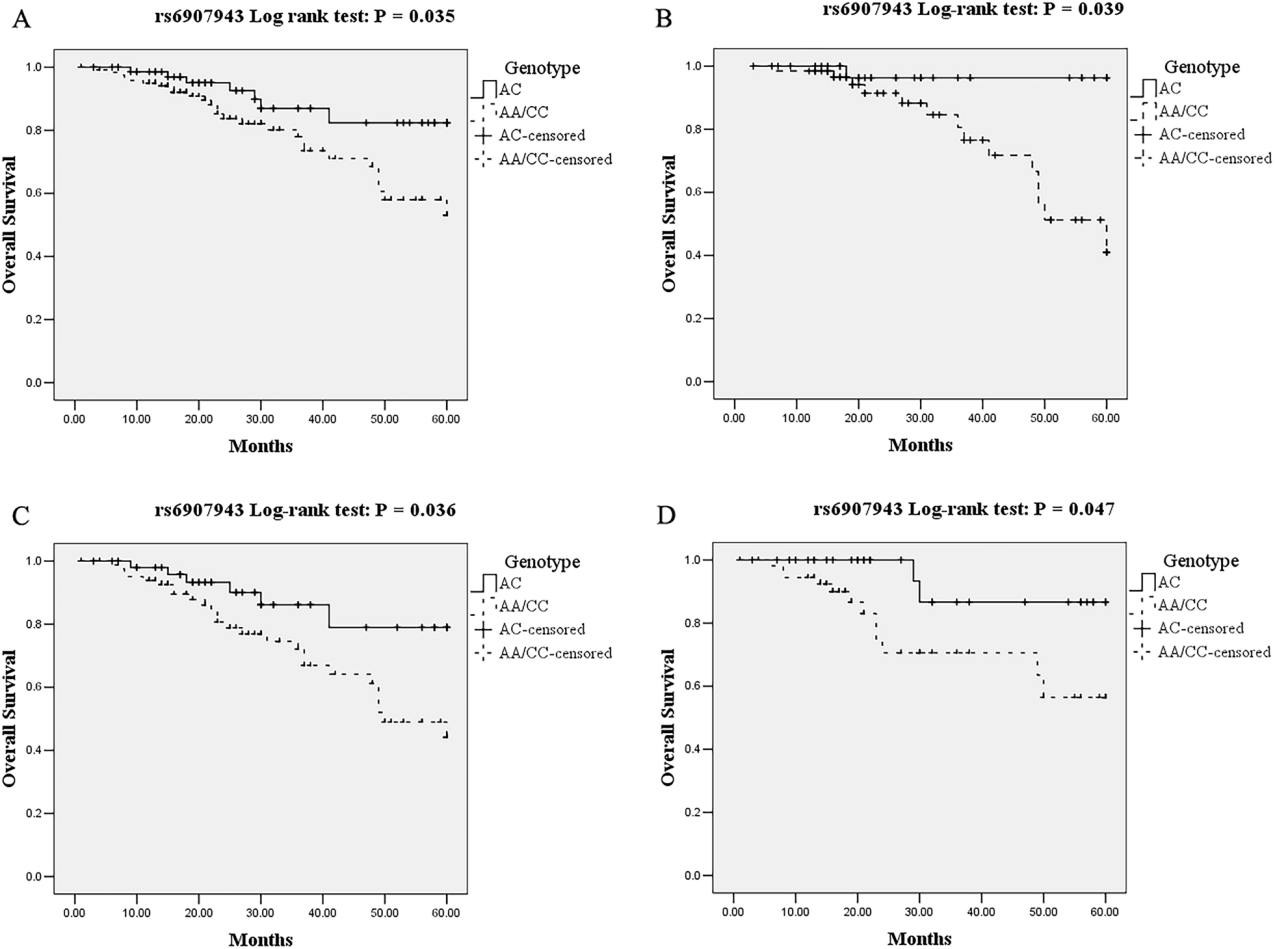
Kaplan-Meier overall survival curves for **A** all of the analyzed EOC patients, **B** early onset EOC patients (age ≤ 50 years), **C** advanced EOC patients (FIGO stage III–IV), and **D** Non-serous EOC patients categorized by tag SNP rs6907943

The associations between *RIPK1* tag SNPs and overall survival of EOC patients were subjected to univariate survival analysis, as well as adjusted by well-known or widely discussed risk factors for survival including age, FIGO stage, histological type, and tumor grade (data shown in Additional file 2: Table S2) in the multiple regression survival analysis (Table 6). Overall survival was analyzed for dependency on genotypes of *RIPK1* tag SNPs using Kaplan-Meier curves. The tag SNP rs6907943 turned out to be a protective factor for overall survival of patients with EOC in univariate survival analysis. Compared with the reference group consisting of rs6907943 homozygotes (AA/CC genotype), the AC heterozygote EOC patients showed a significantly decreased risk for death with HR of 0.41 (95%CI  0.18–0.94, *P* = 0.035) in the over-dominant genetic model. However, results of multivariate regression survival analysis adjusted for age, FIGO stage, histological type, and tumor grade showed that rs6907943 AC heterozygote EOC patients have a decreased risk for death, but not statistically significant (HR = 0.43, 95%CI  0.17–1.05, *P* = 0.064). The overall survival Kaplan-Meier curves for dependency on rs6907943 genotypes in all of the analyzed EOC patients were shown in Fig. 1A.

**Table 6:** Association between the *RIPK1* tag SNPs and overall survival of EOC patients

Characteristics		Overall survival					
		Multivariate survival analysis ^**a**^			Univariate survival analysis		
		HR	95% CI	*P* value	HR	95% CI	*P* value
Model	Genotype						
rs6907943							
Dominant	AA	1	0.25-1.2	0.13	1	0.25-1.06	0.07
	AC/CC	0.54			0.52		
Recessive	AA/AC	1	0.44-5.05	0.53	1	0.46-3.69	0.63
	CC	1.48			1.3		
Overdominant	AA/CC	1	0.17-1.05	0.06	1	**0.18-0.94**	**0.035**
	AC	0.43			**0.41**		
rs9392453							
Dominant	TT	1	0.43-1.91	0.8	1	0.35-1.44	0.35
	CT/CC	0.91			0.71		
Recessive	TT/CT	1	0.04-2.01	0.2	1	0.03-1.65	0.14
	CC	0.27			0.23		
Overdominant	TT/CC	1	0.61-2.76	0.5	1	0.51-2.14	0.91
	CT	1.29			1.04		

Bold value Indicates a significant difference at the 5% level

^**a**^Adjusted for age, FIGO stage, histological type, and tumor grade

We analyzed the influence of *RIPK1* tag SNPs on the overall survival of different EOC patients stratified by age, FIGO stage, histological type, and tumor stage (Additional file 3: Table S3). No association was observed between rs9392453 and overall survival of patients stratified by age, FIGO stage, histological type and tumor stage, or between rs6907943 and overall survival of patients stratified by tumor grade. However, significant influence of rs6907943 on the overall survival of patients stratified by age, FIGO stage, and histological type was identified. In early onset EOC patients (age ≤ 50 years), subjects carrying AC heterozygote have a significantly decreased risk for death compared with those harboring AA/CC homozygote (HR = 0.12, 95%CI  0.02–0.90, *P* = 0.039 for univariate analysis; and HR = 0.12, 95%CI  0.02–0.95, *P* = 0.044 for multivariate analysis adjusted by FIGO stage, histological type, and tumor stage). The overall survival Kaplan-Meier curves for dependency on rs6907943 genotypes in early onset EOC patients (age ≤ 50 years) were shown in Fig. 1B.

In advanced EOC patients (FIGO stage III-IV), subjects with AC heterozygote of rs6907943 had a better clinical outcome compared with those carrying AA/CC homozygote (HR = 0.39, 95%CI  0.16–0.94, *P* = 0.036 for univariate analysis). However, the influence was not significant in multivariate analysis adjusted by age, histological type, and tumor stage (HR = 0.44, 95%CI  0.18–1.09, *P* = 0.076). Figure 1 C showed the overall survival Kaplan-Meier curves for dependency on rs6907943 genotypes in advanced EOC patients (FIGO stage III-IV). The *RIPK1* tag SNP rs6907943 also had a significant influence on the overall survival of EOC patients with non-serious histological type. Patients carrying AC heterozygote had a significant decreased risk for death compared with those harboring AA/CC homozygote (HR = 0.22, 95%CI  0.05–0.98, *P* = 0.047). But the influence was not significant in multivariate analysis adjusted by age, FIGO stage, and tumor stage (HR = 0.21, 95%CI  0.03–1.71, *P* = 0.14). The overall survival Kaplan-Meier curves for dependency on rs6907943 genotypes in non-serous EOC patients were shown in Fig. 1D.

**Fig. 2:**
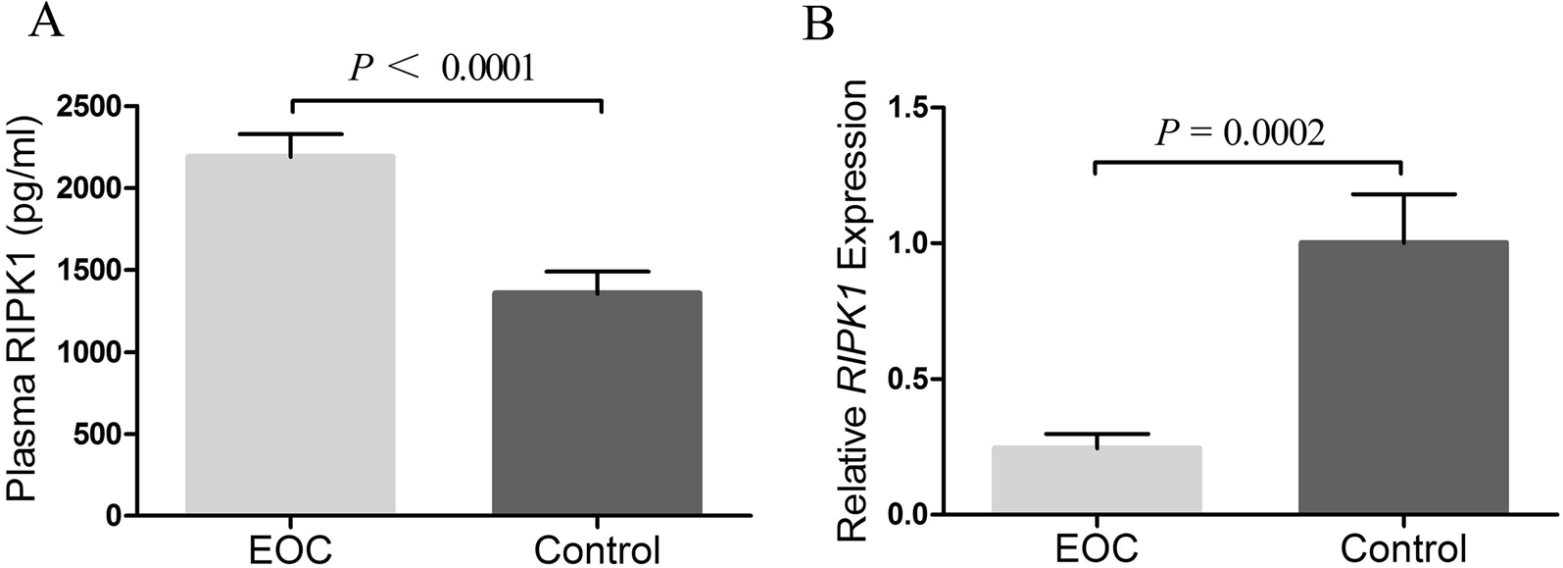
*RIPK1* expression levels in EOC patients and controls (**A**) Plasma RIPK1 levels. **B** RIPK1 mRNA expression in white blood cells. A P value is based on the independent samples *t* test

As shown in Fig. 2 and Additional file 4: Table S4, by analyzing the plasma RIPK1 concentration of 66 EOC patients and 75 controls (data shown in Additional file 5: Table S5), we found RIPK1 levels of patients (2187.96 ± 1142.31 pg/ml) were significantly increased compared with that of controls (1390.94 ± 1140.68 pg/ml) (*P* < 0.0001). However, the *RIPK1* mRNA expression in EOC patients (0.24 ± 0.44) was significantly reduced compared to controls (1 ± 1.57) (*P* = 0.0002). To study the association between genotype and phenotype, plasma RIPK1 concentration and *RIPK1* mRNA expression in patients with respect to *RIPK1* SNPs was investigated. As shown in Additional file 4: Table S4, no significant association between plasma RIPK1 levels, *RIPK1* mRNA expression and genotype of these tag SNPs, patients’ characteristics including age, FIGO stage, histological type or tumor grade was observed.

## Discussion

The impact of *RIPK1* gene tag SNPs, plasma RIPK1 levels and *RIPK1* mRNA expression on the susceptibility to EOC and clinical outcome of these patients was investigated in a Chinese population. The present results revealed that tag SNP rs9392453 was associated with increased EOC risk, but not have any prognostic effect on overall survival of EOC patients. For rs6907943, the CC homozygote was associated with increased risk for early onset of EOC, while AC genotype had a prognostic effect on overall survival of these EOC patients. These two SNPs were not in LD, and haplotype CC (rs6907943–rs9392453) was associated with increased EOC risk. Stratified analysis revealed AC heterozygote of rs6907943 as an independent protective factor for overall survival of early onset EOC patients (age ≤ 50 years). Plasma RIPK1 levels of patients were significantly increased compared with that of controls, conversely, the *RIPK1* mRNA expression in EOC patients was significantly reduced compared to controls. A subset of investigated genes has also presented absence of mRNA-protein correlation, which suggested that the relation between mRNA and protein was not strictly linear, but had a more intrinsic and complex dependence. Different regulation mechanisms (such as synthesis and degradation rates), acting on both the synthesized mRNA and the synthesized protein, may affect the amount of the two molecules differentially [27]. The detection of mRNA expression is accurate but wavelike with time for the maturity and sensitivity of genetic testing technology, and the protein is usually maintaining on a relatively stable expression. Therefore, in our study, the protein expression level presented by ELISA may demonstrate the trending of RIPK1 in vivo, and provide a certain research basis for a potential predictive marker.

The association between genetic variation of *RIPK1* gene and disease including cancers has been reported. The tag SNP of *RIPK1* in Caucasian, rs9391981, was associated with a decreased risk for multiple myeloma in a population residing in Connecticut [21]. Another SNP of *RIPK1*, rs2272990, was significantly associated with both disease-free survival and disease-specific survival of colorectal cancer in a population of Korean, but not associated with prognosis of Korean patients with colorectal cancer treated with capecitabine and oxaliplatin [22, 28]. The SNP rs2272990 was also significantly correlated with time to progression in Korean patients with diffuse large B cell lymphoma treated with R-CHOP [20]. The variations of *RIPK1* have been implicated in the development of Weneger’s granulomatosis in a population of German based on altering apoptosis [29]. However, variations of *RIPK1* was not associated with pancreatic cancer risk in a Canada population, or with risk for non-Hodgkin’s lymphoma in a Caucasian population residing in Minnesota, Iowa, or Wisconsin [30, 31]. According to data of tag SNPs genotyped in the CHB population sample of the HapMap Project (Data Release 24/phaseII, NCBI build 36 assembly, dpSNPb126), both these two widely studied SNPs are in linkage disequilibrium with the tag SNP rs9392453, with the *r*^2^ = 1.0 of correlation between rs9392453 and rs9391981, and the *r*^2^ = 0.849 of correlation between rs9392453 and rs2272990, respectively.

Whole-genome microarrays analysis of uveal melanoma revealed that amplification of *RIPK1* (chromosome 6p) is correlated with better patient survival, but there was no statistically significant correlation between RIPK1 protein express and *RIPK1* gene amplification or patient survival. Results of immunohistochemical assessment for RIPK1 protein showed only a few tumor cells (< 1%) were positive for RIPK1, while dual immunofluorescence to detect RIPK1 and either MITF or MelA showed no concurrent expression of the proteins, suggesting that the RIPK1-expressing cells were not melanoma cells [32]. Recently, it was shown that *RIPK1* gene expression levels of white blood cells enhanced in farmers’ children compared to non-farmers’, and the farm-environmental mediated up-regulation in *RIPK1* contributed partially to their reduced incidence of asthma [33].

RIPK1 is a key mediator of cell death and inflammation. TNF-upregulation related necroptosis mediated by RIPK1 promotes further cell death and neuroinflammation in the pathogenesis of several neurodegenerative diseases including multiple sclerosis, amyotrophic lateral sclerosis (ALS), Parkinson disease and Alzheimer disease [34]. The reduced TAK1 expression produces RIPK1 activation and cooperates with genetic risk factors to promote the onset of ALS [35]. It is reported that inhibition of RIPK1 strongly suppresses inflammation induced by hepatocyte-specific loss of TAK1 [36], and RIPK1 collaborates with TNF receptor-associated factor 2 (TRAF2) to inhibit murine and human hepatocarcinogenesis [37]. In the present study, RIPK1 plasma levels of EOC patients increased, conversely, *RIPK1* mRNA expression in white blood cells of patients decreased, indicating that dysregulated expression of *RIPK1* contributes partially to increased EOC risk. However, the dysregulated expression of *RIPK1*, and the molecular mechanism for *RIPK1* interaction involved in pathogenesis of EOC are needed to further study.

## Conclusions

In conclusion, two tag SNPs of *RIPK1* gene, rs6907943 and rs9392453 were genotyped in a Chinese population of EOC patients and controls in the present study. For the first time, our results indicate that both these tag SNPs are associated with increased risk for early onset of EOC, and SNP rs6907943 is associated with increased overall survival of these early onset EOC patients. Although rs9392453 do not have any prognostic effect on EOC, it is associated with increased susceptibility to EOC. Haplotype analysis showed CC haplotype (rs6907943-rs9392453) is associated with increased EOC susceptibility. RIPK1 plasma levels of EOC patients increased, conversely, *RIPK1* mRNA expression in white blood cells of patients decreased. Nevertheless, the present study may have some limitations. Firstly, the menopausal status, BRCA gene expression, and the adjuvant therapy data of patients were not enrolled for survival analyses, and this might affect the objectivity of our results partly. Further studies enrolled more clinical features and various population could help to establish the true significance of the associations between these tag SNPs and susceptibility to EOC and survival of patients with EOC. The impact of these tag SNPs, and other polymorphisms that are in LD with them, on the initiation, progress, and outcome of EOC, as well as the dysregulated expression of *RIPK1*, especially the molecular mechanism for *RIPK1* involved in pathogenesis of EOC, are needed to further study.

### Supplementary information

** Additional file 1: Table S1.** The results of genotype and clinicopathological data.

** Additional file 2: Table S2.** Genotypes of the SNPs, clinicopathological data and their associations with overall survival of EOC patients.

** Additional file 3: Table S3.**  Association between the RIPK1 tag SNPs and overall survival of different groups of EOC patients.

** Additional file 4: Table S4.**  Relationship between plasma levels of RIPK1, RIPK1 mRNA expression and patients’ characteristics.

** Additional file 5: Table S5.** Plasma RIPK1 concentration, RIPK1 mRNA expression and clinicopathological data.

## Data Availability

The datasets supporting the conclusions of this article are included within the article and its additional files.
