# Supplementary material for: RIPK1 polymorphisms and expression levels: impact on genetic susceptibility and clinical outcome of epithelial ovarian cancer
Source: Cancer Cell Int. 2023 Nov 23;23:290. doi: 10.1186/s12935-023-03139-7 (PMC10668399; doi:10.1186/s12935-023-03139-7)
Supplement: Supplementary file 3 — Additional file 3: Table S3. Association between the RIPK1 tag SNPs and overall survival of different groups of EOC patients. [file 12935_2023_3139_MOESM3_ESM.doc]

**Table S3.** Association between the *RIPK1* tag SNPs and overall survival of different groups of EOC patients

| Characteristics | |  | Overall survival ^a^ | | | | | | | | | | | | | | |
| --- | --- | --- | --- | --- | --- | --- | --- | --- | --- | --- | --- | --- | --- | --- | --- | --- | --- |
|  |  |  | Multivariate survival analysis **^b^** | | |  | Univariate survival analysis | | |  | Multivariate survival analysis **^b^** | | |  | Univariate survival analysis | | |
|  | |  | HR | 95% CI | *P* value |  | HR | 95% CI | *P* value |  | HR | 95% CI | *P* value |  | HR | 95% CI | *P* value |
| **rs6907943** |  |  | | | | | | | |  |  | | | | | | |
| Model | Genotype |  | Age ≤ 50 | | | | | | |  | Age > 50 | | | | | | |
| Dominant | AA |  | 1 |  |  |  | 1 |  |  |  | 1 |  |  |  | 1 |  |  |
|  | AC/CC |  | 0.33 | 0.10-1.13 | 0.08 |  | 0.49 | 0.17-1.45 | 0.2 |  | 0.60 | 0.21-1.72 | 0.35 |  | 0.56 | 0.21-1.47 | 0.56 |
| Recessive | AA/AC |  | 1 |  |  |  | 1 |  |  |  | 1 |  |  |  | 1 |  |  |
|  | CC |  | 1.79 | 0.44-7.24 | 0.42 |  | 2.57 | 0.80-8.23 | 0.11 |  | NA | NA | 0.99 |  | 0.05 | NA-743.65 | 0.53 |
| Overdominant | AA/CC |  | 1 |  |  |  | 1 |  |  |  | 1 |  |  |  | 1 |  |  |
|  | AC |  | **0.12** | **0.02-0.95** | **0.044** |  | **0.12** | **0.02-0.9** | **0.039** |  | 0.72 | 0.25-2.07 | 0.55 |  | 0.67 | 0.26-1.77 | 0.42 |
|  |  |  |  |  |  |  |  |  |  |  |  |  |  |  |  |  |  |
|  |  |  | FIGO stage: Ⅰ-Ⅱ | | | | | | |  | FIGO stage: Ⅲ-Ⅳ | | | | | | |
| Dominant | AA |  | 1 |  |  |  | 1 |  |  |  | 1 |  |  |  | 1 |  |  |
|  | AC/CC |  | 0.24 | NA | 0.78 |  | 0.96 | 0.06-16.02 | 0.98 |  | 0.56 | 0.25-1.25 | 0.16 |  | 0.53 | 0.25-1.11 | 0.09 |
| Recessive | AA/AC |  | 1 |  |  |  | 1 |  |  |  | 1 |  |  |  | 1 |  |  |
|  | CC |  | 0.12 | NA | 0.87 |  | 0.04 | NA | 0.74 |  | 1.52 | 0.44-5.23 | 0.51 |  | 1.45 | 0.51-4.15 | 0.49 |
| Overdominant | AA/CC |  | 1 |  |  |  | 1 |  |  |  | 1 |  |  |  | 1 |  |  |
|  | AC |  | 0.03 | NA | 0.85 |  | 1.56 | 0.1-25.53 | 0.75 |  | 0.44 | 0.18-1.09 | 0.076 |  | **0.39** | **0.16-0.94** | **0.036** |
|  |  |  |  |  |  |  |  |  |  |  |  |  |  |  |  |  |  |
|  |  |  | Histological type: Serous | | | | | | |  | Histological type: Non-serous | | | | | | |
| Dominant | AA |  | 1 |  |  |  | 1 |  |  |  | 1 |  |  |  | 1 |  |  |
|  | AC/CC |  | 0.83 | 0.33-2.10 | 0.7 |  | 0.74 | 0.3-1.8 | 0.5 |  | 0.29 | 0.03-2.53 | 0.23 |  | 0.28 | 0.08-1.02 | 0.054 |
| Recessive | AA/AC |  | 1 |  |  |  | 1 |  |  |  | 1 |  |  |  | 1 |  |  |
|  | CC |  | 1.56 | 0.44-5.51 | 0.49 |  | 1.3 | 0.38-4.44 | 0.68 |  | NA | NA | 0.99 |  | 1.26 | 0.17-9.66 | 0.82 |
| Overdominant | AA/CC |  | 1 |  |  |  | 1 |  |  |  | 1 |  |  |  | 1 |  |  |
|  | AC |  | 0.64 | 0.23-1.79 | 0.4 |  | 0.6 | 0.22-1.66 | 0.33 |  | 0.21 | 0.03-1.71 | 0.14 |  | **0.22** | **0.05-0.98** | **0.047** |
|  |  |  |  |  |  |  |  |  |  |  |  |  |  |  |  |  |  |
|  |  |  | Tumor grade: G1-G2 | | | | | | |  | Tumor grade: G3 | | | | | | |
| Dominant | AA |  | 1 |  |  |  | 1 |  |  |  | 1 |  |  |  | 1 |  |  |
|  | AC/CC |  | NA | NA | 0.34 |  | 0.82 | 0.07-9.10 | 0.87 |  | 0.59 | 0.26-1.34 | 0.21 |  | 0.52 | 0.23-1.18 | 0.12 |
| Recessive | AA/AC |  | 1 |  |  |  | 1 |  |  |  | 1 |  |  |  | 1 |  |  |
|  | CC |  | 2.12 | 0.08-60.13 | 0.66 |  | 6.48 | 0.41-103.82 | 0.19 |  | 1.18 | 0.27-5.10 | 0.83 |  | 0.83 | 0.2-3.52 | 0.81 |
| Overdominant | AA/CC |  | 1 |  |  |  | 1 |  |  |  | 1 |  |  |  | 1 |  |  |
|  | AC |  | NA | NA | 0.64 |  | 0.03 | NA | 0.51 |  | 0.52 | 0.21-1.29 | 0.16 |  | 0.51 | 0.2-1.25 | 0.14 |
|  |  |  |  |  |  |  |  |  |  |  |  |  |  |  |  |  |  |
| **rs9392453** |  |  |  |  |  |  |  |  |  |  |  |  |  |  |  |  |  |
|  |  |  | Age ≤ 50 | | | | | | |  | Age > 50 | | | | | | |
| Dominant | TT |  | 1 |  |  |  | 1 |  |  |  | 1 |  |  |  | 1 |  |  |
|  | CT/CC |  | 0.48 | 0.15 | 1.51 |  | 0.53 | 0.18-1.55 | 0.24 |  | 1.03 | 0.38-2.77 | 0.96 |  | 0.85 | 0.22-2.16 | 0.85 |
| Recessive | TT/CT |  | 1 |  |  |  | 1 |  |  |  | 1 |  |  |  | 1 |  |  |
|  | CC |  | 0.4 | 0.05-3.06 | 0.4 |  | 0.41 | 0.05-3.1 | 0.39 |  | NA | NA | 0.98 |  | 0.04 | NA | 0.39 |
| Overdominant | TT/CC |  | 1 |  |  |  | 1 |  |  |  | 1 |  |  |  | 1 |  |  |
|  | CT |  | 0.67 | 0.19-2.35 | 0.53 |  | 0.72 | 0.23-2.25 | 0.72 |  | 1.37 | 0.51-3.69 | 0.53 |  | 1.22 | 0.48-3.11 | 0.68 |
|  |  |  |  |  |  |  |  |  |  |  |  |  |  |  |  |  |  |
|  |  |  | FIGO stage: Ⅰ-Ⅱ | | | | | | |  | FIGO stage: Ⅲ-Ⅳ | | | | | | |
| Dominant | TT |  | 1 |  |  |  | 1 |  |  |  | 1 |  |  |  | 1 |  |  |
|  | CT/CC |  | 116.25 | NA | 0.74 |  | 1.85 | 0.12-29.63 | 0.66 |  | 0.79 | 0.37-1.70 | 0.55 |  | 0.62 | 0.30-1.29 | 0.2 |
| Recessive | TT/CT |  | 1 |  |  |  | 1 |  |  |  | 1 |  |  |  | 1 |  |  |
|  | CC |  | 0.7 | NA | 0.99 |  | 0.04 | NA | 0.74 |  | 0.27 | 0.04-1.99 | 0.2 |  | 0.26 | 0.04-1.92 | 0.19 |
| Overdominant | TT/CC |  | 1 |  |  |  | 1 |  |  |  | 1 |  |  |  | 1 |  |  |
|  | CT |  | 6826.61 | NA | 0.85 |  | 3.35 | 0.21-54.10 | 0.4 |  | 1.14 | 0.52-2.49 | 0.74 |  | 0.85 | 0.4-1.81 | 0.68 |
|  |  |  |  |  |  |  |  |  |  |  |  |  |  |  |  |  |  |
|  |  |  | Histological type: Serous | | | | | | |  | Histological type: Non-serous | | | | | | |
| Dominant | TT |  | 1 |  |  |  | 1 |  |  |  | 1 |  |  |  | 1 |  |  |
|  | CT/CC |  | 1.08 | 0.44-2.66 | 0.87 |  | 1.11 | 0.46-2.69 | 0.82 |  | 0.32 | 0.09-1.16 | 0.08 |  | 0.33 | 0.09-1.20 | 0.09 |
| Recessive | TT/CT |  | 1 |  |  |  | 1 |  |  |  | 1 |  |  |  | 1 |  |  |
|  | CC |  | NA | NA | 0.98 |  | 0.04 | NA | 0.3 |  | 0.56 | 0.07-4.29 | 0.58 |  | 0.58 | 0.08-4.47 | 0.6 |
| Overdominant | TT/CC |  | 1 |  |  |  | 1 |  |  |  | 1 |  |  |  | 1 |  |  |
|  | CT |  | 1.68 | 0.67-4.2 | 0.27 |  | 1.84 | 0.76-4.46 | 0.18 |  | 0.33 | 0.07-1.47 | 0.15 |  | 0.33 | 0.08-1.50 | 0.15 |
|  |  |  |  |  |  |  |  |  |  |  |  |  |  |  |  |  |  |
|  |  |  | Tumor grade: G1-G2 | | | | | | |  | Tumor grade: G3 | | | | | | |
| Dominant | TT |  | 1 |  |  |  | 1 |  |  |  | 1 |  |  |  | 1 |  |  |
|  | CT/CC |  | NA | NA | 0.33 |  | 0.82 | 0.07-9.09 | 0.87 |  | 1.07 | 0.49-2.36 | 0.86 |  | 0.99 | 0.45-2.12 | 0.98 |
| Recessive | TT/CT |  | 1 |  |  |  | 1 |  |  |  | 1 |  |  |  | 1 |  |  |
|  | CC |  | NA | NA | NA |  | NA | NA | NA |  | 0.34 | 0.05-2.56 | 0.3 |  | 0.27 | 0.04-2.01 | 0.2 |
| Overdominant | TT/CC |  | 1 |  |  |  | 1 |  |  |  | 1 |  |  |  | 1 |  |  |
|  | CT |  | NA | NA | 0.33 |  | 0.82 | 0.07-9.09 | 0.87 |  | 1.44 | 0.65-3.20 | 0.37 |  | 1.46 | 0.67-3.18 | 0.34 |

**^a^** 66 patients with missing survival data removed.

**^b^** Adjusted for age, FIGO stage, histological type, and tumor grade.
