# Supplementary material for: RIPK1 polymorphisms and expression levels: impact on genetic susceptibility and clinical outcome of epithelial ovarian cancer
Source: Cancer Cell Int. 2023 Nov 23;23:290. doi: 10.1186/s12935-023-03139-7 (PMC10668399; doi:10.1186/s12935-023-03139-7)
Supplement: Supplementary file 4 — Additional file 4: Table S4. Relationship between plasma levels of RIPK1, RIPK1 mRNA expression and patients’ characteristics. [file 12935_2023_3139_MOESM4_ESM.doc]

**Table S4.** Relationship between plasma levels of RIPK1, *RIPK1* mRNA expression and patients’ characteristics.

| Variable analyzed |  |  | Plasma RIPK1 (pg/ml) | | | | |  | *RIPK1* mRNA relative expression | | | | |
| --- | --- | --- | --- | --- | --- | --- | --- | --- | --- | --- | --- | --- | --- |
|  | Total, *N* |  | Mean | SD | SEM | Median | *P* |  | Mean | SD | SEM | Median | *P* |
| Case-control |  |  |  |  |  |  |  |  |  |  |  |  |  |
| Controls | 75 |  | 1390.94 | 1140.68 | 111.85 | 1039.22 | **< 0.0001** |  | 1 | 1.57 | 0.18 | 0.25 | **0.0002** |
| EOC | 66 |  | 2187.96 | 1142.31 | 140.61 | 1960.49 |  |  | 0.24 | 0.44 | 0.05 | 0.12 |  |
| Age |  |  |  |  |  |  |  |  |  |  |  |  |  |
| ≤50 | 39 |  | 2211.80 | 1031.36 | 165.15 | 2183.63 | 0.84 |  | 0.33 | 0.56 | 0.09 | 0.14 | 0.06 |
| ＞50 | 27 |  | 2153.52 | 1305.92 | 251.32 | 1778.21 |  |  | 0.12 | 0.11 | 0.02 | 0.09 |  |
| FIGO stage |  |  |  |  |  |  |  |  |  |  |  |  |  |
| Ⅰ-Ⅱ | 18 |  | 1939.54 | 791.10 | 186.46 | 1946.68 | 0.28 |  | 0.25 | 0.3 | 0.07 | 0.21 | 0.91 |
| Ⅲ-Ⅳ | 48 |  | 2281.11 | 1243.28 | 179.45 | 1960.49 |  |  | 0.24 | 0.49 | 0.07 | 0.1 |  |
| Histological type |  |  |  |  |  |  |  |  |  |  |  |  |  |
| Serous | 43 |  | 2251.82 | 1182.39 | 180.31 | 1915.03 | 0.54 |  | 0.25 | 0.51 | 0.08 | 0.1 | 0.97 |
| Non-serous | 23 |  | 2068.57 | 1078.61 | 224.91 | 2030.88 |  |  | 0.24 | 0.29 | 0.06 | 0.14 |  |
| Tumor grade ^a^ |  |  |  |  |  |  |  |  |  |  |  |  |  |
| G1-G2 | 12 |  | 1748.39 | 620.28 | 179.06 | 1771.49 | 0.15 |  | 0.23 | 0.5 | 0.07 | 0.1 | 0.73 |
| G3 | 46 |  | 2305.18 | 1264.94 | 186.51 | 2018.41 |  |  | 0.18 | 0.17 | 0.05 | 0.12 |  |
| rs6907943 |  |  |  |  |  |  |  |  |  |  |  |  |  |
| AA | 33 |  | 2150.56 | 1298.75 | 226.08 | 1713.01 | 0.58 |  | 0.23 | 0.41 | 0.07 | 0.1 | 0.83 |
| AC | 28 |  | 2311.38 | 1001.77 | 189.32 | 2149.39 |  |  | 0.28 | 0.52 | 0.1 | 0.13 |  |
| CC | 5 |  | 1743.63 | 755.73 | 337.97 | 2005.95 |  |  | 0.16 | 0.11 | 0.05 | 0.18 |  |
| rs9392453 |  |  |  |  |  |  |  |  |  |  |  |  |  |
| TT | 37 |  | 2146.58 | 1228.33 | 201.94 | 1773.72 | 0.52 |  | 0.23 | 0.4 | 0.07 | 0.13 | 0.78 |
| TC | 27 |  | 2304.44 | 1037.47 | 199.66 | 2168.64 |  |  | 0.28 | 0.52 | 0.1 | 0.16 |  |
| CC ^b^ | 2 |  | 1380.82 | 884.06 | 625.13 | 1380.82 |  |  | 0.06 | 0.07 | 0.05 | 0.06 |  |

*N* corresponds to the number of individuals.

^a^ 8 patients with missing tumor grade value removed
